# Prevalence Factors Associated With Vision Impairment and Blindness Among Individuals 85 Years and Older in Russia

**DOI:** 10.1001/jamanetworkopen.2021.21138

**Published:** 2021-08-17

**Authors:** Mukharram M. Bikbov, Gyulli M. Kazakbaeva, Ellina M. Rakhimova, Iuliia A. Rusakova, Albina A. Fakhretdinova, Azaliia M. Tuliakova, Songhomitra Panda-Jonas, Timur R. Gilmanshin, Rinat M. Zainullin, Natalia I. Bolshakova, Kamilia R. Safiullina, Ainur V. Gizzatov, Ildar P. Ponomarev, Dilya F. Yakupova, Nail E. Baymukhametov, Nikolay A. Nikitin, Jost B. Jonas

**Affiliations:** 1 Ufa Eye Research Institute, Ufa, Bashkortostan, Russia; 2 Privatpraxis Jonas and Panda-Jonas, Heidelberg, Germany; 3 Department of Ophthalmology, Medical Faculty Mannheim, Heidelberg University, Mannheim, Germany; 4 Institute of Molecular and Clinical Ophthalmology, Basel, Switzerland

## Abstract

**Importance:**

Visual performance is important for quality of life. However, vision impairment among adults 85 years and older has not been intensively examined.

**Objective:**

To assess the prevalence of mild vision impairment (VI), moderate to severe vision impairment, and blindness and the factors associated with these conditions among a population 85 years and older.

**Design, Setting, and Participants:**

The Ural Very Old Study was a population-based cohort study conducted in rural and urban areas in Bashkortostan, Russia from 2017 to 2020. Among 1882 eligible individuals 85 years and older, 1526 participants (81.1%) were enrolled.

**Exposures:**

Ophthalmologic, physical, and mental examinations.

**Main Outcomes and Measures:**

Prevalence of vision impairment and blindness based on best-corrected visual acuity (BCVA; measured using modified Early Treatment of Diabetic Retinopathy Study charts) in the better eye or both eyes. Mild vision impairment was defined as BCVA worse than 6/12 to 6/18, and moderate to severe VI was defined as BCVA worse than 6/18 but equal to or better than 3/60. Blindness was defined as BCVA worse than 3/60.

**Results:**

Among 1526 participants, 1149 individuals (75.3%; 846 women [73.6%]; mean [SD] age, 88.2 [2.8 years]) had available BCVA measurements and were included in the present analysis. Mild vision impairment was present in 114 individuals (9.9%; 95% CI, 8.2%-11.7%), moderate to severe VI in 562 individuals (48.9%; 95% CI, 46.0%-51.8%), and blindness in 68 individuals (5.9%; 95% CI, 4.6%-7.3%). Factors associated with moderate to severe VI were cataracts (324 individuals [57.7% of those with moderate to severe VI and 28.2% of total population; 95% CI, 25.6%-30.8%]), secondary cataracts (4 individuals [0.7% of those with moderate to severe VI and 0.3% of total population; 95% CI, 0%-0.7%]), age-related macular degeneration (78 individuals [13.9% of those with moderate to severe VI and 6.8% of total population; 95% CI, 5.3%-8.3%]), glaucoma (45 individuals [8.0% of those with moderate to severe VI and 3.9% of total population; 95% CI, 2.8%-5.0%]), corneal opacifications (26 individuals [4.6% of those with moderate to severe VI and 2.3% of total population; 95% CI, 1.4%-3.1%]), myopic maculopathy (13 individuals [2.3% of those with moderate to severe VI and 1.1% of total population; 95% CI, 0.5%-1.7%]), and nonglaucomatous optic nerve damage (4 individuals [0.7% of those with moderate to severe VI and 0.3% of total population; 95% CI, 0%-0.7%]). Factors associated with blindness were cataracts (33 individuals [48.5% of those with blindness and 2.9% of total population; 95% CI, 1.9%-3.8%]), age-related macular degeneration (15 individuals [22.1% of those with blindness and 1.3% of total population; 95% CI, 0.7%-2.0%]), glaucoma (7 individuals [10.3% of those with blindness and 0.6% of total population; 95% CI, 0.2%-1.1%]), myopic maculopathy (3 individuals [4.4% of those with blindness and 0.3% of total population; 95% CI, 0%-0.6%]), and corneal opacifications (2 individuals [2.9% of those with blindness and 0.2% of total population; 95% CI, 0%-0.4%]). Higher moderate to severe VI prevalence was associated with older age (odds ratio [OR], 1.19; 95% CI, 1.11-1.28; *P* < .001), higher blood pressure (OR, 1.01; 95% CI, 1.00-1.02; *P* = .03), lower hand grip force (OR, 0.88; 95% CI, 0.83-0.95; *P* < .001), lower score on the Mini-Mental State Examination (OR, 0.95; 95% CI, 0.92-0.98; *P* < .001), lower prothrombin index (OR, 0.93; 95% CI, 0.89-0.97; *P* < .001), lower refractive error (OR, 0.91; 95% CI, 0.85-0.97; *P* = .006), and lower prevalence of previous cataract surgery (OR, 0.48; 95% CI, 0.33-0.68; *P* < .001).

**Conclusions and Relevance:**

In this cohort study of individuals 85 years and older, the prevalence of moderate to severe VI and blindness was relatively high. Cataracts were the main reversible condition associated with vision loss, and age-related macular degeneration, glaucoma, and myopic maculopathy were the main irreversible conditions. Because a higher prevalence of moderate to severe VI was associated with lower cognitive function and physical strength, improvement of vision through increases in cataract surgery and measures to prevent and treat irreversible conditions may help to improve cognitive function and physical strength.

## Key Points

**Question:**

What is the prevalence of moderate to severe vision impairment (VI) and blindness among individuals 85 years and older in Russia, and what are the factors associated with moderate to severe VI and blindness?

**Findings:**

In this cohort study of 1526 Russian adults 85 years and older, the prevalence of moderate to severe VI and blindness was 49% and 6%, respectively; moderate to severe VI and blindness were associated with cataracts, age-related macular degeneration, glaucoma, and myopic maculopathy. A higher prevalence of moderate to severe VI and blindness was significantly associated with lower physical strength (measured by hand grip force) and lower cognitive function (measured by Mini-Mental State Examination score).

**Meaning:**

This study found a relatively high prevalence of VI and blindness among adults 85 years and older, with cataracts as the primary reversible condition associated with vision loss; these findings suggest that increases in cataract surgery may be helpful to address the lower cognitive function and physical strength associated with VI and blindness.

## Teaser

This cohort study assesses the prevalence of mild vision impairment, moderate to severe vision impairment, and blindness among individuals 85 years and older in Russia and examines the factors associated with moderate to severe vision impairment and blindness.

## Introduction

Visual performance is important for an individual’s quality of life and public health in general.^1,2^ Recent meta-analyses^3,4^ estimated that 43.3 million individuals were blind, and 295 million individuals had moderate to severe vision impairment (VI) in 2020. The most common factors associated with blindness in individuals 85 years and older were cataracts, glaucoma, undercorrected refractive error, age-related macular degeneration (AMD), and diabetic retinopathy.^4^ These and other meta-analyses were limited by a scarcity of population-based studies focusing on adults 85 years and older. Most available studies had an age-related inclusion criterion of 40 years and older, with fewer than 5% of study participants 85 years and older. Our hypothesis was that the prevalence of vision impairment and blindness and their associated factors may differ in a population of individuals 85 years and older compared with younger populations. Therefore, we conducted the Ural Very Old Study (UVOS) to examine the prevalence of vision impairment and blindness and the factors associated with these conditions in a population 85 years and older, to explore the association of vision impairment and blindness with other demographic and clinical variables, and to compare these data with findings obtained among a younger population in the same study regions.

## Methods

The UVOS was a population-based study performed in Kirovskii, a rural region that is 65 km from the capital of Ufa, and the Republic of Bashkortostan, Russia, an urban region of Ufa that is approximately 1300 km east of Moscow. The study was conducted between November 2017 and December 2020 and was approved by the ethics committee of the Academic Council of the Ufa Eye Research Institute. Written informed consent was obtained from all participants. This study followed the Strengthening the Reporting of Observational Studies in Epidemiology (STROBE) reporting guideline for cohort studies and the Guidelines for Accurate and Transparent Health Estimates Reporting (GATHER).^5,6^

Individuals who were 85 years or older and living in the study regions were eligible for inclusion. Among 1882 eligible individuals, 1526 participants (81.1%) were enrolled in the study. Participants in the urban study region included the inhabitants of 3 small private retirement homes; there were no retirement homes in the rural study region. The participation rate did not substantially vary between the urban group (1238 of 1523 individuals [81.3%]) and the rural group (288 of 359 individuals [80.2%]). According to the 2010 Russian census, the sex and age composition of the study population was consistent with that of the Russian population who were 85 years and older, with a preponderance of women.^7,8^

All participants received a standardized interview conducted by trained social workers; this interview comprised almost 300 questions on socioeconomic background, self-reported ethnic background, diet, smoking, physical activity, quality of life, quality of vision, history of any type of injury or injury associated with interpersonal violence, health assessment, medical history, previous neurologic events, cognitive function, and hearing loss.^9,10,11,12,13,14^ Physical and clinical examinations included measurement of anthropomorphic parameters, arterial blood pressure and pulse rate, dynamometric assessment of handgrip strength, and biochemical examination of blood samples obtained under fasting conditions. Arterial hypertension was defined based on recommendations from the American College of Cardiology and the American Heart Association in 2017.^15^ Diabetes was characterized as a fasting glucose concentration of ≥126 mg/dL (to convert to mmol/L, multiply by 0.0555) or a self-reported history of physician diagnosis of diabetes or drug treatment for diabetes. Cognitive function was assessed using the Mini-Mental State Examination (MMSE),^11^ anxiety was measured using the State-Trait Anxiety Inventory,^16^ and depression was assessed using the Center for Epidemiologic Studies–Depression scale.^9^ The estimated glomerular filtration rate was calculated using the equation developed by the Chronic Kidney Disease Epidemiology Collaboration.^17^ The design of the UVOS was similar to that of the Ural Eye and Medical Study (UEMS), which has been described in detail previously.^18,19^

The ophthalmologic examinations comprised the measurement of presenting, uncorrected, and best-corrected visual acuity (BCVA) using modified Early Treatment of Diabetic Retinopathy Study charts; automated refractometry; static perimetry; anterior segment imaging using a Scheimflug camera (Pentacam HR, Typ 70900; OCULUS); sonographic biometric measurement of axial length; slit lamp biomicroscopy of the anterior and posterior ocular segment; noncontact tonometry; photography of the cornea and lens (slit lamp and camera; Topcon Corp); photography of the optic disc (VISUCAM 500; Carl Zeiss Meditec) and macula (Smartscope EY4; Optomed); and spectral-domain optical coherence tomography of the optic nerve head (RS-3000 Advance; NIDEK) and macula (DRI OCT Triton plus; Topcon Corp). The guidelines published by the Beckman Initiative for Macular Research Classification Committee^20^ were applied for the definition of AMD (with no AMD defined as no visible drusen or pigmentary abnormalities, normal aging changes [with no increased risk of developing late AMD] defined as the presence of small drusen [<63 μm], early AMD defined as the presence of medium drusen [≥63 μm to <125 μm] but no pigmentary abnormalities, intermediate AMD defined as the presence of large drusen [≥125 μm] or pigmentary abnormalities, and late AMD defined as the presence of lesions associated with neovascular AMD or geographic atrophy). Glaucoma was defined using morphological criteria described by Foster et al.^21^ Based on criteria from the World Health Organization,^22^ we defined mild vision impairment as BCVA worse than 6/12 to 6/18 in the better eye or both eyes, moderate to severe VI as BCVA worse than 6/18 but equal to or better than 3/60 in the better eye or both eyes, and blindness as BCVA worse than 3/60 in the better eye or both eyes.

### Statistical Analysis

We assessed the prevalence of blindness, moderate to severe VI, and mild VI (reported as means with 95% CIs) using SPSS for Windows, version 25.0 (SPSS Institute), and we performed a binary regression analysis of the association between the prevalence of moderate to severe VI or blindness and other demographic, clinical, and ocular factors.^23^ This analysis was followed by a multivariable regression analysis, with the prevalence of blindness or moderate to severe VI as the dependent variable and all factors that were significantly associated with prevalence as independent variables in the univariate analyses. We then removed, in a step-by-step manner, those factors from the list of independent variables that had collinearity with other factors and that were no longer significantly associated with the prevalence of moderate to severe VI or blindness. We calculated the odds ratios (ORs) and their 95% CIs. The significance threshold was 2-sided *P* < .05.

## Results

Among 1526 participants in the UVOS, 1149 individuals (75.3%; 846 women [73.6%]; 303 men [26.4%]; mean [SD] age, 88.2 [2.8 years]) with available BCVA measurements who received examinations in the hospital were included in the present analysis. A total of 408 individuals (35.5%) were Russian, 504 (43.9%) were Tatar, 135 (11.7%) were Bashkir, 44 (3.8%) were Chuvash, 7 (0.6%) were Mari, and 51 (4.4%) were of other ethnicities. The mean (SD) axial length was 23.1 (1.1) mm (range, 19.4-28.9 mm). The prevalence of moderate myopia (axial length, 24.5 to <26.5 mm) was 47 of 717 individuals (6.6%; 95% CI, 4.7%-8.4%), and the prevalence of high myopia (axial length, ≥26.5 mm) was 10 of 717 individuals (1.4%; 95% CI, 0.5%-2.3%). Those who received BCVA assessments were significantly younger than those who did not (mean [SD] age, 88.2 [2.8] years vs 88.8 [3.2] years; *P* = .001), whereas those with vs without BCVA assessments did not differ significantly in educational level (mean [SD], 4.5 [2.1] years vs 4.6 [2.0] years; *P* = .19) and sex (303 men and 846 women vs 87 men and 290 women; *P* = .22).

Among 1149 participants, 39 individuals (3.4%) were illiterate. A total of 236 individuals (20.5%) completed fifth grade, 249 (21.7%) completed eighth grade, 48 (4.2%) completed tenth grade, and 35 (3.0%) completed eleventh grade; 241 individuals (21.0%) had some specialized secondary education, 285 (24.8%) were college graduates, and 4 (0.3%) were postgraduates. A total of 199 individuals (17.3%) were living in a joint family (defined as extended family comprising parents, their children, and the children’s spouses and offspring in 1 household), 95 (8.3%) were living with nuclear family, 441 (38.4%) were living alone, and 405 (35.2%) were living with 1 family member; 250 individuals (21.8%) were married, 22 (81.9%) were unmarried, 21 (1.8%) were divorced, and 847 (73.7%) were widowed. Almost all of the participants owned a home or apartment (1115 individuals [97.0%]) and a television and/or telephone (1054 individuals [91.7%]). The mean (SD) body height was 157 (9) cm, and the mean (SD) body mass index (calculated as weight in kilograms divided by height in meters squared) was 26.6 (4.5). Of 1149 participants, 114 individuals (9.9%; 95% CI, 8.2%-11.7%) met the criteria for having mild VI, 562 individuals (48.9%; 95% CI, 46.0%-51.8%) met the criteria for having moderate to severe VI in the better eye or both eyes, and 68 individuals (5.9%; 95% CI, 4.6%-7.3%) met the criteria for having blindness in the better eye or both eyes (Table 1).

**Table 1.** Prevalence of Mild Vision Impairment (MVI), Moderate to Severe Vision Impairment (MSVI), and Blindness Stratified by Age and Sex

Age group, y	No.	MVI^a^		MSVI^b^		Blindness^c^	
		No. (%)^d^	95% CI	No. (%)^d^	95% CI	No. (%)^d^	95% CI
All							
85-86	401	43 (10.7)	7.7-13.8	157 (39.2)	34.4-44.0	18 (4.5)	2.5-6.5
87-88	303	33 (10.9)	7.4-14.4	151 (49.8)	44.2-55.5	13 (4.3)	2.0-6.6
89-90	225	22 (9.8)	5.9-13.7	113 (50.2)	43.6-56.8	16 (7.1)	3.7-10.5
91-92	134	8 (6.0)	1.9-10.0	87 (64.9)	56.7-73.1	9 (6.7)	2.4-11.0
≥93	86	8 (9.3)	3.0-15.6	54 (62.8)	52.4-73.2	12 (14.0)	6.5-21.4
Men							
85-86	110	7 (6.4)	1.7-11.0	42 (38.2)	29.0-47.4	3 (2.7)	0-5.8
87-88	79	11 (13.9)	6.1-21.7	35 (44.3)	33.1-55.5	1 (1.3)	0-3.8
89-90	61	6 (9.8)	2.2-17.5	34 (55.7)	42.9-68.6	5 (8.2)	1.1-15.3
91-92	27	2 (7.4)	0-18.0	16 (59.3)	39.5-79.1	2 (7.4)	0-18.0
≥93	26	0	NA	18 (69.2)	50.2-88.2	4 (15.4)	0.5-30.3
Women							
85-86	291	36 (12.4)	8.6-16.2	115 (39.5)	33.9-45.2	15 (5.2)	2.6-7.7
87-88	224	22 (9.8)	5.9-13.8	116 (51.8)	45.2-58.4	12 (5.4)	2.4-8.3
89-90	164	16 (9.8)	5.2-14.4	79 (48.2)	40.4-55.9	11 (6.7)	2.8-10.6
91-92	107	6 (5.6)	1.2-10.0	71 (66.4)	57.3-75.5	7 (6.5)	1.8-11.3
≥93	60	8 (13.3)	4.5-22.2	36 (60.0)	47.2-72.8	8 (13.3)	4.5-22.2

Abbreviation: NA, not applicable.

^a^ Mild vision impairment was defined as best-corrected visual acuity worse than 6/12 to 6/18 in the better eye or both eyes (measured by modified Early Treatment of Diabetic Retinopathy Study charts).

^b^ Moderate to severe vision impairment was defined as best-corrected visual acuity worse than 6/18 but equal to or better than 3/60 in the better eye or both eyes (measured by modified Early Treatment of Diabetic Retinopathy Study charts).

^c^ Blindness was defined as best-corrected visual acuity worse than 3/60 in the better eye or both eyes (measured by modified Early Treatment of Diabetic Retinopathy Study charts).

^d^ Percentages calculated across rows.

In the univariate analysis, the prevalence of moderate to severe VI increased with various demographic, clinical, and ocular factors, including older age (OR, 1.13; 95% CI, 1.08-1.18; *P* < .001) and rural region of residence (OR, 1.89; 95% CI, 1.44-2.49; *P* < .001) (Figure; Table 2). In the multivariable analysis, after removing parameters owing to collinearity and lack of statistical significance, the final model revealed that a higher prevalence of moderate to severe VI was associated with older age (OR, 1.19; 95% CI, 1.11-1.28; *P* < .001), higher mean blood pressure (OR, 1.01; 95% CI, 1.00-1.02; *P* = .03), lower score on the Mini-Mental State Examination (OR, 0.95; 95% CI, 0.92-0.98; *P* < .001), more substantial cylindrical refractive error (OR, 0.63; 95% CI, 0.54-0.73; *P* < .001), lower prevalence of previous cataract surgery (OR, 0.48; 95% CI, 0.33-0.68; *P* < .001), lower prothrombin index (OR, 0.93; 95% CI, 0.89-0.97; *P* < .001), and lower hand grip force (OR, 0.88; 95% CI, 0.83-0.95; *P* < .001) (Table 3). When the parameter of cylindrical refractive error was replaced by refractive error (spherical equivalent), the latter was significantly associated with higher moderate to severe VI prevalence (OR, 0.91; 95% CI, 0.85-0.97; *P* = .006). When refractive error was replaced by axial length, the latter was significantly associated with moderate to severe VI prevalence (OR, 1.25; 95% CI, 1.02-1.53; *P* = .03). When MMSE score was removed, higher moderate to severe VI prevalence was associated with a higher depression score (OR, 1.02; 95% CI, 1.01-1.04; *P* = .007).

**Figure.**
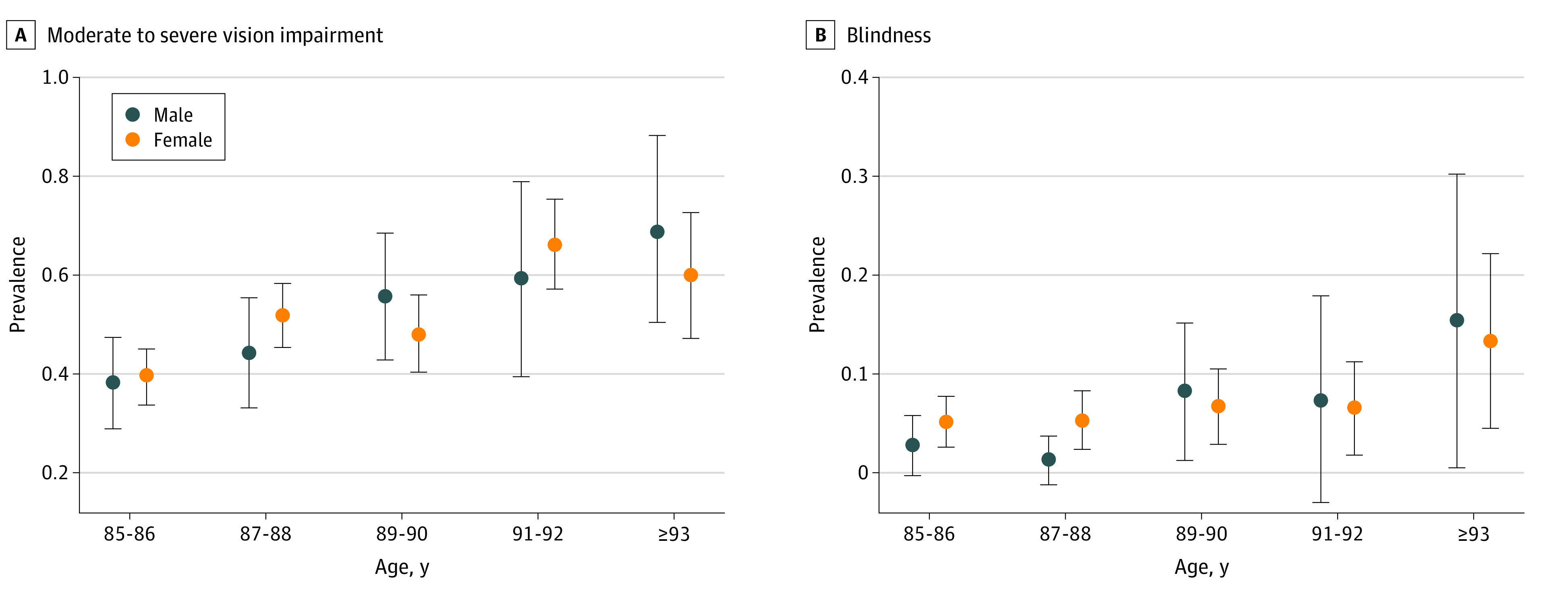
Prevalence of Moderate to Severe Vision Impairment and Blindness Stratified by Age and Sex A, Moderate to severe vision impairment was defined as visual acuity worse than 6/18 but equal to or better than 3/60 in the better eye or both eyes. B, Blindness was defined as visual acuity worse than 3/60 in the better eye or both eyes. Circles represent prevalence, and error bars represent 95% CIs.

**Table 2.** Univariate Analysis of Association Between Participant Characteristics and Prevalence of Moderate to Severe Vision Impairment (MSVI) and Blindness

Characteristic	MSVI		Blindness	
	OR (95% CI)	*P* value	OR (95% CI)	*P* value
Age, y	1.13 (1.08-1.18)	<.001	1.16 (1.07-1.25)	<.001
Sex	1.06 (0.82-0.18)	.67	1.28 (0.71-2.31)	.41
Region of residence (urban vs rural)	1.89 (1.44-2.49)	<.001	2.06 (1.24-3.42)	<.001
Ethnicity (non-Russian vs Russian)	0.91 (0.72-1.16)	.46	1.20 (0.73-1.98)	.48
Height	1.01 (1.00-1.03)	.12	1.00 (0.97-1.03)	.95
BMI	0.99 (0.96-1.02)	.46	0.95 (0.89-1.02)	.16
Waist-to-hip ratio	0.73 (0.18-2.92)	.66	0.08 (0.004-1.41)	.08
Educational level	0.88 (0.83-0.93)	<.001	0.84 (0.74-0.95)	.005
Current smoking	0.96 (0.24-3.83)	.95	1.02 (NA)	>.99
Any alcohol consumption	0.64 (0.44-0.93)	.02	0.71 (0.30-1.68)	.44
Daily meals, No.	0.89 (0.77-1.03)	.13	0.58 (0.40-0.83)	.003
Fruit consumption, d/wk	0.91 (0.86-0.97)	.002	0.94 (0.84-1.06)	.34
Vegetable consumption, d/wk	0.90 (0.83-0.97)	.004	0.87 (0.76-1.00)	.05
History of headaches	1.20 (1.02-1.63)	.03	1.22 (0.75-1.99)	.43
History of dementia	0.94 (0.60-1.48)	.94	3.47 (1.81-6.65)	<.001
Cholesterol level, mg/dL				
HDL	0.80 (0.69-0.93)	.004	0.54 (0.37-0.79)	.001
Total	0.93 (0.85-1.02)	.11	0.70 (0.56-0.87)	.001
Triglyceride level, mg/dL	0.99 (0.84-1.16)	.87	0.42 (0.25-0.71)	.001
International normalized ratio	0.98 (0.39-2.46)	.98	19.3 (3.50-106.00)	.001
Prothrombin time, %	1.00 (0.98-1.01)	.42	0.96 (0.94-0.99)	.002
Erythrocyte level, ×10^6^/μL	0.79 (0.63-0.99)	.04	0.90 (0.56-1.46)	.67
Prevalence of diabetes	1.42 (1.01-2.00)	.04	1.11 (0.55-2.23)	.77
Blood pressure				
Systolic	1.01 (1.00-1.01)	.003	1.00 (0.99-1.01)	.93
Diastolic	1.01 (1.00-1.02)	.004	1.01 (1.00-1.03)	.12
Arterial hypertension stage	1.15 (1.02-1.29)	.02	1.07 (0.83-1.37)	.61
Hearing loss score	1.02 (1.01-1.03)	<.001	1.02 (1.00-1.04)	.02
CES-D score	1.02 (1.01-1.03)	.003	1.05 (1.03-1.08)	<.001
STAI score	1.01 (1.00-1.03)	.03	1.03 (1.01-1.06)	.005
Manual dynamometry				
Right hand	0.98 (0.96-0.99)	.004	0.90 (0.87-0.94)	<.001
Left hand	0.97 (0.96-0.99)	.003	0.90 (0.86-0.95)	<.001
MMSE score	0.95 (0.93-0.97)	<.001	0.89 (0.85-0.93)	<.001
Refractive error (spherical equivalent), diopter	0.93 (0.89-0.98)	.005	1.03 (0.90-1.18)	.69
Axial length, mm	1.08 (0.95-1.24)	.24	0.85 (0.56-1.27)	.42
Intraocular pressure^a^	1.06 (1.03-1.10)	<.001	1.01 (0.98-1.03)	.63
Cataract surgery	0.70 (0.49-0.98)	.04	0.52 (0.41-0.67)	<.001

Abbreviations: BMI, body mass index (calculated as weight in kilograms divided by height in meters squared); CES-D, Center for Epidemiologic Studies–Depression scale; HDL, high-density lipoprotein; MMSE, Mini-Mental State Examination; NA, not available; OR, odds ratio; STAI, State-Trait Anxiety Inventory.

^a^ Assessed using Goldmann applanation tonometry.

**Table 3.** Multivariable Analysis of Association Between Participant Characteristics and Prevalence of Moderate to Severe Vision Impairment (MSVI), Blindness, and Combined MSVI and Blindness

Characteristic	MSVI		Blindness		Combined MSVI and blindness	
	OR (95% CI)	*P* value	OR (95% CI)	*P* value	OR (95% CI)	*P* value
Age, y	1.19 (1.11-1.28)	<.001	1.17 (1.04-1.31)	.01	1.20 (1.11-1.29)	<.001
Mean blood pressure, mm Hg	1.01 (1.00-1.02)	.03	NA	NA	1.02 (1.00-1.03)	.01
Mini Mental Test score	0.95 (0.92-0.98)	<.001	0.92 (0.87-0.97)	.001	0.93 (0.90-0.96)	<.001
Previous cataract surgery	0.48 (0.33-0.68)	<.001	NA	NA	0.49 (0.34-0.71)	<.001
Refractive error (spherical equivalent), diopter	0.91 (0.85-0.97)	.006	NA	NA	NA	NA
Cylindrical refractive error, negative diopter^a^	0.63 (0.54-0.73)	<.001	NA	NA	0.63 (0.54-0.73)	<.001
Dynamometric hand grip, daN	0.88 (0.83-0.95)	<.001	NA	NA	NA	NA
Prothrombin index, %	0.93 (0.89-0.97)	<.001	NA	NA	NA	NA

Abbreviations: NA, not applicable (not included in the final model of this multivariable analysis); OR, odds ratio.

^a^ Cylindrical refractive error was used in lieu of spherical equivalent.

In the univariate analysis, the prevalence of blindness increased with demographic, clinical, and ocular factors, such as older age (OR, 1.16; 95% CI, 1.07-1.25; *P* < .001) and rural region of residence (OR, 2.06; 95% CI, 1.24-3.42; *P* < .001) (Table 2). In the multivariable model, a higher prevalence of blindness was associated with older age (OR, 1.17; 95% CI, 1.04-1.31; *P* = .01) and lower MMSE score (OR, 0.92; 95% CI, 0.87-0.97; *P* = .001) (Table 3). The prevalence of combined moderate to severe VI and blindness was associated with older age (OR, 1.20; 95% CI, 1.11-1.29; *P* < .001), higher mean blood pressure (OR, 1.02; 95% CI, 1.00-1.03; *P* = .01), lower MMSE score (OR, 0.93; 95% CI, 0.90-0.96; *P* < .001), lower prevalence of previous cataract surgery (OR, 0.49; 95% CI, 0.34-0.71; *P* < .001), and more substantial cylindrical refractive error (OR, 0.63; 95% CI, 0.54-0.73; *P* < .001) (Table 3). In contrast, a higher MMSE score was associated with younger age (standardized β = −0.16; nonstandardized β = −0.35 [95% CI, −0.50 to −0.20]; *P* < .001), higher educational level (standardized β = 0.27; nonstandardized β = 0.81 [95% CI, 0.60-1.03]; *P* < .001), urban region of residence (standardized β = 0.11; nonstandardized β = 1.57 [95% CI, 0.52-2.63]; *P* = .004), lower prevalence of moderate to severe VI and blindness (standardized β = −0.13; nonstandardized β = −1.62 [95% CI, −2.46 to −0.78]; *P* < .001), and lower hearing loss score (standardized β = −0.09; nonstandardized β = −0.03 [95% CI, −0.06 to −0.01]; *P* = .01).

The factors associated with moderate to severe VI were cataracts (324 participants [57.7% of those with moderate to severe VI and 28.2% of total population; 95% CI, 25.6%-30.8%]), secondary cataracts (4 participants [0.7% of those with moderate to severe VI and 0.3% of total population; 95% CI, 0%-0.7%]), AMD (78 participants [13.9% of those with moderate to severe VI and 6.8% of total population; 95% CI, 5.3%-8.3%]), glaucoma (45 participants [8.0% of those with moderate to severe VI and 3.9% of total population; 95% CI, 2.8%-5.0%]), corneal opacifications (26 participants [4.6% of those with moderate to severe VI and 2.3% of total population; 95% CI, 1.4%-3.1%]), myopic maculopathy (13 participants [2.3% of those with moderate to severe VI and 1.1% of total population; 95% CI, 0.5%-1.7%]), nonglaucomatous optic nerve damage (4 participants [0.7% of those with moderate to severe VI and 0.3% of total population; 95% CI, 0%-0.7%]), macular edema of unknown origin (2 participants [0.4% of those with moderate to severe VI and 0.2% of total population; 95% CI, 0%-0.4%]), macular holes (1 participant [0.2% of those with moderate to severe VI and 0.1% of total population; 95% CI, 0%-0.3%]), and unclear reason (65 participants [11.6% of those with moderate to severe VI and 5.7% of total study population; 95% CI, 4.3%-7.0%]) (Table 4). Differentiating between reversible (eg, cataracts, secondary cataracts, corneal opacifications, macular edema, and macular holes) and irreversible (eg, AMD, glaucoma, myopic maculopathy, and nonglaucomatous optic nerve damage) factors associated with vision loss, 357 of 1149 individuals (31.1%) had reversible moderate to severe VI and 140 of 1149 individuals (12.2%) had irreversible moderate to severe VI.

**Table 4.** Factors Associated With Moderate to Severe Vision Impairment (MSVI) and Blindness Stratified by Age and Sex

Factor	Participants with disease-associated MSVI, No.	Proportion of participants with disease-associated MSVI, No. (%) (n = 562)^a^	Prevalence of disease-associated MSVI, No. (%) [95% CI] (n = 1149)^b^	Participants with disease-associated blindness, No.	Proportion of participants with disease-associated blindness, No. (%) (n = 68)^c^	Prevalence of disease-associated blindness, No. (%) [95% CI] (n = 1149)^b^
Cataract	324	324 (57.7)	324 (28.2) [25.6-30.8]	33	33 (48.5)	33 (2.9) [1.9-3.8]
Secondary cataract	4	4 (0.7)	4 (0.3) [0-0.7]	0	0	0
Age-related macular degeneration	78	78 (13.9)	78 (6.8) [5.3-8.3]	15	15 (22.1)	15 (1.3) [0.7-2.0]
Myopic maculopathy	13	13 (2.3)	13 (1.1) [0.5-1.7]	3	3 (4.4)	3 (0.3) [0-0.6]
Glaucoma	45	45 (8.0)	45 (3.9) [2.8-5.0]	7	7 (10.3)	7 (0.6) [0.2-1.1]
Nonglaucomatous optic nerve damage	4	4 (0.7)	4 (0.3) [0-0.7]	0	0	0
Macular hole	1	1 (0.2)	1 (0.1) [0-0.3]	0	0	0
Macular edema of unknown origin	2	2 (0.4)	2 (0.2) [0-0.4]	0	0	0
Corneal scar	26	26 (4.6)	26 (2.3) [1.4-3.1]	2	2 (2.9)	2 (0.2) [0-0.4]
Unclear	65	65 (11.6)	65 (5.7) [4.3-7.0]	8	8 (11.8)	8 (0.7) [0.2-1.2]

^a^ Proportion among all participants with MSVI, which was defined as best-corrected visual acuity worse than 6/18 but equal to or better than 3/60 in the better eye or both eyes (measured by modified Early Treatment of Diabetic Retinopathy Study charts).

^b^ Prevalence in total study population.

^c^ Proportion among all participant with blindness, which was defined as best-corrected visual acuity worse than 3/60 in the better eye or both eyes (measured by modified Early Treatment of Diabetic Retinopathy Study charts).

The factors associated with blindness were cataracts (33 participants [48.5% of those with blindness and 2.9% of total population; 95% CI, 1.9%-3.8%]), AMD (15 participants [22.1% of those with blindness and 1.3% of total population; 95% CI, 0.7%-2.0%]), glaucoma (7 participants [10.3% of those with blindness and 0.6% of total population; 95% CI, 0.2%-1.1%]), myopic maculopathy (3 participants [4.4% of those with blindness and 0.3% of total population; 95% CI, 0%-0.6%]), corneal opacifications (2 participants [2.9% of those with blindness and 0.2% of total population; 95% CI, 0%-0.4%]), and unclear reason (8 participants [11.8% of those with blindness and 0.7% of total study population; 95% CI, 0.2%-1.2%]) (Table 4). Among all participants, 33 individuals (2.9%) had reversible blindness, and 25 (2.2%) had irreversible blindness .

## Discussion

In this cohort study of an ethnically mixed population 85 years and older from Bashkortostan, Russia, the prevalence of blindness, moderate to severe VI, and mild VI was 5.9%, 48.9%, and 9.9%, respectively. The most common factors associated with blindness and moderate to severe VI were cataracts (48.5% and 57.7%, respectively), AMD (22.1% and 13.9%), and glaucoma (10.3% and 8.0%).

The findings of the present study cannot be directly compared with observations made in other studies because previous investigations typically did not include a sufficient number of participants who were 85 years and older. In the UEMS, which was performed using a similar study design in the same study regions among a population 40 years and older (mean [SD] age, 59.0 [10.7] years; range, 40-94 years), the prevalence of blindness, moderate to severe VI, and mild VI was 0.19%, 3.1%, and 3.1%, respectively.^18,19^ The marked difference in prevalence was mainly because of the substantial difference in participant age. Among participants in the oldest age category in the UEMS (202 participants ≥80 years; mean [SD] age, 83.0 [2.9] years), the prevalence of moderate to severe VI was 19.2% (95% CI, 10.3%-28.2%) among men and 27.6% (95% CI, 19.6%-35.7%) among women^23^; this finding was substantially lower than the prevalence of 48.9% (95% CI, 46.0%-51.8%) found in the older population in the present study (mean [SD] age, 88.2 [2.8] years). In the ALIENOR study^24^ of individuals 75 years and older, which used the oldest age inclusion criterion among ophthalmologically related population-based studies to date, the prevalence of blindness and moderate to severe VI has not yet been reported. In other population-based studies of younger populations in high-income countries,^25,26^ the age-standardized prevalence of blindness and moderate to severe VI in 2015 was 0.15% (95% CI, 0.06%-0.26%) and 1.27% (95% CI, 0.55%-2.17%), respectively,^25^ whereas in rural central India, among a population 30 years and older, the prevalence of blindness and moderate to severe VI from 2008 to 2009 was 0.5% (95% CI, 0.3%-0.7%) and 7.0% (95% CI, 6.3%-7.8%), respectively.^26^ In the latter study, the prevalence of moderate to severe VI and blindness increased to 39.0% and 5.4%, respectively, among individuals 80 years and older.^26^

In our study, as in other previous investigations,^4,23,25,26,27,28,29^ the presence of cataracts was the most common reversible condition associated with moderate to severe VI and blindness, followed by irreversible conditions associated with vision loss, including AMD, glaucoma, myopic maculopathy, and corneal opacifications. None of the individuals with vision impairment or blindness had diabetic retinopathy as the main condition associated with vision loss (Table 4). In a recent meta-analysis of data from the Global Burden of Disease Study conducted by the Vision Loss Expert Group,^4^ the most common factors associated with blindness worldwide were cataracts (15.2 million individuals), glaucoma (3.6 million individuals), undercorrected refractive error (2.3 million individuals), AMD (1.8 million individuals), and diabetic retinopathy (0.9 million individuals), whereas the most common factors associated with moderate to severe VI were undercorrected refractive error (86.1 million individuals) and cataracts (78.8 million individuals). Differentiated into global regions, the lists of the most common factors associated with blindness and moderate to severe VI^4,23,25,26,27,28,29^ do not differ markedly from those reported in the present study. The relatively high prevalence of cataracts as factors associated with blindness (2.9% of our total study population) and moderate to severe VI (28.2% of our total study population) suggests the need to increase the rate of cataract surgery in the study regions.

In the global meta-analysis,^4^ myopic maculopathy was not separately assessed regarding its frequency as a factor associated with moderate to severe VI and blindness. In our study, myopic maculopathy was associated with blindness in 4.4% of all participants with blindness (12.0% of participants with irreversible blindness) and with moderate to severe VI in 2.3% of all participants with moderate to severe VI (9.3% of participants with irreversible moderate to severe VI) (Table 4). The prevalence of myopic maculopathy as a factor associated with blindness and moderate to severe VI in our total study population was 0.3% (95% CI, 0%-0.6%) and 1.1% (95% CI, 0.5%-1.7%), respectively.

Myopic maculopathy was the third most common factor associated with irreversible blindness and moderate to severe VI. In the UEMS population, which included participants with substantially younger ages, myopic maculopathy was the second most common factor associated with irreversible blindness (2 of 8 individuals [25.0%] with irreversible blindness, or 2 of 5893 individuals [0.03%] in the total study population) and the second most common factor associated with irreversible moderate to severe VI (11 of 73 individuals [15.1%] with irreversible moderate to severe VI, or 11 of 5893 individuals [0.2%] in the total study population).^23^ The prevalence of myopic maculopathy as a factor associated with blindness (0.3% in our study vs 0.03% in the UEMS) and moderate to severe VI (1.1% in our study vs 0.2% in the UEMS) was thus higher in our older population compared with the younger population of the UEMS. Even if one assumes a further age-related increase in the prevalence of myopic maculopathy, the 5- to 10-fold higher prevalence of myopic maculopathy as a factor associated with blindness or moderate to severe VI in our older study population compared with the middle-aged population of the UEMS suggests that, in the UEMS and UVOS study regions, the prevalence of myopic maculopathy as a factor associated with moderate to severe VI or blindness (in the middle-aged population) may not have substantially increased compared with previous periods.^30^ The importance of myopic maculopathy as a factor associated with moderate to severe VI and blindness has also been reported in population-based studies conducted in East Asia,^27,28,29^ such as the Beijing Eye Study,^27^ in which myopic maculopathy was the most common factor associated with irreversible vision impairment and blindness in a population 40 years and older who were examined in 2001.

Factors associated with moderate to severe VI and blindness were older age, lower MMSE score, lower dynamometric hand grip force, lower prothrombin index, lower prevalence of previous cataract surgery, and more substantial cylindrical refractive error (Table 3). Although the associations with older age have also been reported in previous studies,^4,23,25,26,27,28,29^ the association between a higher prevalence of moderate to severe VI and blindness and lower cognitive function (as measured by MMSE score) is of interest because another study^31^ found an association between the prevalence of Alzheimer disease and hearing loss. This finding suggests that cataract surgery (and the provision of best-correcting glasses), in particular, may be a means of reducing the risk of cognitive dysfunction. The finding is also consistent with the observation of lower hand grip force among participants with visual disabilities in our study. Hand grip strength has been regarded as a biomarker of concurrent overall strength, upper limb function, bone mineral density, depression, multimorbidity, and quality of life.^32^

### Strengths and Limitations

This study has strengths. To our knowledge, the study is the first population-based investigation of the prevalence of moderate to severe VI and blindness among individuals 85 years and older to include a relatively large sample and many demographic, clinical, and ocular factors.

This study also has limitations. First, the presenting visual acuity of participants was not assessed, so we could not determine the prevalence of undercorrection of refractive error as a factor associated with vision impairment and blindness. Second, dense cataracts prevented examination of the fundus; thus, any additional disorders of the optic nerve and macula were not detected. This limitation might have produced an underestimation of the prevalence of macular and optic nerve diseases. Third, difficulty with access to medical services may have been the primary factor associated with the relatively high prevalence of moderate to severe VI and blindness in the study population. We did not, however, assess the interval between the participants’ last ophthalmologic examinations and the start of the study.

## Conclusions

Among a population 85 years and older from Bashkortostan, Russia, the prevalence of moderate to severe VI and blindness was 48.9% and 5.9%, respectively, with 28.2% and 2.9% of the total study population experiencing moderate to severe VI or blindness associated with cataracts. Because cataracts were the main reversible condition associated with vision loss, increases in cataract surgery may be helpful to address blindness and VI among participants in the study region. Age-related macular degeneration was associated with moderate to severe VI and blindness in 3.9% and 0.6% of the population, respectively, and myopic maculopathy was the third most common factor associated with irreversible blindness and moderate to severe VI. Efforts may be intensified for the prevention and treatment of these conditions. Blindness and moderate to severe VI were associated with lower cognitive function and physical strength (as measured by hand grip force); thus, addressing vision impairment through measures such as cataract surgery may also help to improve cognitive function and physical strength.
